# Infection prevention and control measures in audiology practice within public healthcare facilities in KwaZulu-Natal province, South Africa

**DOI:** 10.4102/sajcd.v66i1.636

**Published:** 2019-12-02

**Authors:** Nasim B. Khan, Chenay R. Charles, Naedene Naidoo, Amanda Nokubonga, Ndabenhle A. Mkhwanazi, Hella M.T.E. Moustache

**Affiliations:** 1 Discipline of Audiology, School of Health Sciences, University of KwaZulu-Natal, Durban, South Africa

**Keywords:** infection prevention, infection control, handwashing, universal precautions, personal protective equipment, waste management

## Abstract

**Background:**

Audiologists have a clinical and ethical responsibility to create a working environment, designed to reduce the potential for cross-contamination or transmission of infections.

**Objectives:**

To describe the infection prevention and control (IPC) measures utilised and the opinions of audiologists and speech therapists, and audiologists (A/STAs) towards IPC in public healthcare facilities in KwaZulu-Natal province, South Africa.

**Method:**

A quantitative, descriptive survey was utilised and entailed completing an online questionnaire. The Cronbach’s alpha (0.82) indicated good internal consistency of the tool. Forty-nine A/STAs from 29 public healthcare facilities responded.

**Results:**

Most participants (82%) followed a generic Department of Health policy on IPC, while 67% alluded to a discipline-specific policy. Participants had received training in infection control but indicated that further instruction was required for audiology-specific infection control procedures. Only 57% indicated that they ‘sometimes’ wore gloves with every patient during direct clinical contact. An association between the healthcare facility level and the wearing of gloves was found to be statistically significant (*p* = 0.025). Participants at regional and tertiary levels contended that gloves should be worn during most procedures versus those at district levels of care. While 96% washed their hands after each patient, only 76% washed their hands before each patient. Twenty-nine per cent indicated that they only ‘sometimes’ wore masks when in contact with patients with communicable diseases. Approximately one-third disinfected touch surfaces and toys, based on the clinician’s discretion. The majority (86%) of participants, however, always followed the correct protocol for medical waste disposal. Despite training and the availability of policies, some practitioners displayed poor IPC practices in terms of universal precautions, personal protective equipment, handwashing and sterilisation.

**Conclusion:**

Further education, training and awareness related to appropriate IPC measures are recommended for audiologists. It is envisaged that this will lead to more effective IPC measures in audiology practice thereby reducing the risk of infection transmission.

## Introduction

Infection prevention and control (IPC) measures are defined as eliminating or minimising the potential spread of disease by consciously managing the clinical environment (Bankaitis, 2005a). According to the World Health Organization (WHO), infectious diseases are the second leading cause of death, after heart diseases, worldwide (WHO, 2008). Nosocomial infections or hospital acquired infections (HAIs) can result in prolonged hospital stays, increased mortality and morbidity, and an increased financial burden for the health sector (Mahomed, Mahomed, Sturm, Knight, & Moodley, 2017). Despite the limited literature and lack of intervention and impact studies, the clinical environment in patient care has widely been recognised as crucial for patient safety through proper IPC measures (Peters et al., 2018).

The scope of audiology has expanded over the years to include vestibular and balance testing, intraoperative monitoring and cerumen management; therefore, infection control is an area that has become increasingly important (Burco, 2007). Audiologists provide rehabilitative and diagnostic services that are required by patients who differ in various factors, including underlying disease, age and socio-economic status (Ehlert & Naude, 2014). Healthy patients have a general resistance to infection, whereas neonatal intensive care unit (NICU) infants and the elderly, who also require audiology services, have lower resistance and may be more susceptible to infections. Immunocompromised individuals with underlying diseases, that is, tuberculosis (TB), human immunodeficiency virus (HIV) and acquired immunodeficiency syndrome (AIDS), may present with a hearing loss because of the nature of the virus and exposure to antiretroviral treatment (Burco, 2007).

Audiologists in clinical practice have a great degree of indirect and direct contact with patients during procedures to assess the auditory system (Health Professionals Council of South Africa [HPCSA], 2012). Although the risk of HIV and AIDS transmission is remote, by conducting various procedures, including but not limited to, vestibular testing and intraoperative monitoring, audiologists may be exposed to bodily fluids and blood, thus increasing the risk of infection (Burco, 2007). The majority of HAIs occur via the transmission of pathogens, especially by healthcare practitioners (HCPs) who do not wash their hands after treating a patient or properly complying with hospital IPC measures (Adegboye, Zakari, Ahmed, & Olufemi, 2018). The hands of HCPs are thus responsible for up to 50% – 70% of all HAIs and are the main vectors for spreading diseases (Peters et al., 2018).

According to the WHO, there were approximately 558 000 cases of multidrug-resistant (MDR) TB worldwide in 2017 (WHO, 2018). South Africa has one of the world’s highest TB epidemics, which is driven by HIV (Claassens et al., 2013; Peters et al., 2018) and the second highest number of identified MDR TB cases after India (Churchyard et al., 2014). Its treatment includes a group of antibiotics termed aminoglycosides, which are ototoxic and target the cochlea vestibular system. Managing individuals with TB requires the inclusion of the appropriate HCPs, including audiologists, who are essential in monitoring the effects of ototoxic medication on hearing (Khoza-Shangase, 2013). A South African study by Khoza-Shangase and Van Rie (2017) conducted with 96 participants from an HIV and AIDS research unit indicated that, of those diagnosed with HIV and AIDS, 14% reported co-occurring vestibular symptoms and 69% reported experiencing co-occurring audiological symptoms. Therefore, audiologists and speech therapists, and audiologists (A/STAs) in a South African context have numerous contacts with patients with communicable diseases and should implement effective IPC practices when in contact with all patients (Khoza-Shangase & Van Rie, 2017), thus embracing universal precautions.

In a study by Alp, Leblebicioglu, Doganay and Voss (2011) regarding infection control practices in countries with limited resources, a comparison was made between two countries, Turkey and the Netherlands, of different socio-economic statuses. The results indicated that the attitude of HCPs in hospitals that were not well equipped affected the infection rate. Alp et al.’s study findings correlated with Peters et al.’s (2018) study that low salaries, inadequate facilities and limited infrastructure caused HCPs to lose enthusiasm for their profession and thus do the bare minimum. Therefore, this may have resulted in the incorrect implementation of IPC measures (Alp et al., 2011; Sahiledengle, Gebresilassie, Getahun, & Hiko, 2018).

The HPCSA recommends that general universal infection control measures need to be followed by all HCPs, regarding hygiene and cleanliness (HPCSA, 2002). These routine precautions include: (1) washing hands before and after every patient; (2) wearing gloves when conducting an oral peripheral examination; (3) washing, wiping or soaking any non-disposable equipment, including toys, with sterilising solution, whenever used; and (4) the correct disposal of waste in appropriate waste disposal bags directly after use (HPCSA, 2002). This is reiterated by the South African Speech-Language Hearing Association (SASLHA) (2011) that indicates that hand hygiene, handwashing, the use of personal protective equipment (PPE) and cleaning or sterilising and disinfecting are essential procedures in audiology-specific infection control for minimising the spread of infectious diseases (SASLHA, 2011). Similarities were noted in Ehlert and Naude’s (2014) and Burco’s (2007) studies, which indicated that 84% of respondents from Burco’s study and 82% from Ehlert and Naude’s study recognised the significance of handwashing. Results from a study by Archanalakshmi, Stanly and Paul (2015) revealed that when individuals worked for 30 h – 40 h, only 62% indicated that they would wash hands. However, only 22% of individuals who worked for 40 h – 50 h would wash hands. This indicates that when HCPs work for longer hours, the chances of washing hands decrease (Archanalakshmi et al., 2015). Gloves can be used to reduce hand contamination from blood and other infectious agents, but cannot prevent injury from sharp objects or instruments. The risk of cross-contamination increases if gloves are not changed between patients or if they are contaminated with bodily fluids. Their use is not a substitute for handwashing, therefore necessitating that hands be washed after the removal of gloves (WHO, 2001, 2004).

According to the Canadian Association of Speech, Language Pathologists and Audiologists (CASLPA, 2010), audiologists use and reuse clinical equipment across a wide range of patients. These consist of otoscope speculae, probes and headphones, which can be reused, but should be cleaned, disinfected and/or sterilised after each patient. Cleaning eliminates gross contamination but does not remove germs from the objects or surfaces and should therefore be a precursor to disinfection and sterilisation (CASLPA, 2010). Results from Ehlert and Naude’s study indicated that 60% of respondents sterilised their audiological equipment after seeing each patient. Less than 50% of respondents disinfected toys and touch surfaces, compared to Burco’s study where approximately 40% disinfected, and would only do so if there was visible contamination, with the latter disinfection dictated by the audiologists’ discretion (Burco, 2007; Ehlert & Naude, 2014). Toys that are situated in the waiting area should also be disinfected daily, as children could insert them into their mouths, resulting in saliva on the toy, which is a source of bacteria (Burco, 2007; Ehlert & Naude, 2014). Thus, the significance of infection control regarding toys, audiological equipment and touch surfaces before and after patients should be emphasised, as this reduces the transmission of diseases and cross-contamination of microorganisms. Correct waste disposal is a strategy used to prevent and minimise infections among HCPs and community members, which is an essential infection control intervention (Haifete, Justus, & Iita, 2016). If medical waste is not disposed of correctly, it can become an agent to increase the spread of diseases such as HIV and AIDS, hepatitis B and other communicable diseases, which may result in HCPs, patients and even the community being affected (Haifete et al., 2016).

Limited studies have been conducted in South Africa in this area, despite the emerging burden of communicable or infectious diseases and the expanding scope of practice in audiology. In addition, audiologists working within the public sector face various resource limitations, which could have an impact on the IPC measures available. Information obtained from the study will be used to make contextually appropriate recommendations for infection control measures and practices.

## Materials and methods

The main aim of the study was to determine the IPC measures utilised by A/STAs in public healthcare facilities in KwaZulu-Natal (KZN). A secondary aim was to describe the opinions of A/STAs towards IPC in public healthcare facilities in KZN province, South Africa. The objectives of the study were to describe the IPC policies used, to describe the opinions of A/STAs regarding the training received and training needed, to describe the A/STAs’ handwashing practices, to determine the personal protective measures available and used by A/STAs for IPC and to determine the opinions of A/STAs regarding equipment management and waste disposal in public healthcare facilities in KZN, South Africa.

A quantitative, non-experimental, descriptive survey research design was used to obtain information from the 83 A/STAs conveniently sampled from 39 public healthcare facilities in the 11 districts within KZN during 2017. A pilot study was conducted on 9 of the 83 A/STAs, with the remaining 74 A/STAs being the target group for the main study. Responses were obtained from 59% (*n* = 49 people), this being from 29 out of 39 of the targeted public healthcare facilities. A questionnaire (Appendix 1) was developed from the three studies conducted by Amlani (1999), Burco (2007) and Ehlert and Naude (2014) consisting of five sections (A–E) with 40 questions. The questionnaire included multiple-choice, closed, open-ended and contingency questions (Leedy & Ormrod, 2015).

The A/STAs were emailed the link to the Google forms survey where an informed consent letter provided information about the research study, assurance of confidentiality and instructions for completing the questionnaire. After 10 working days, the researchers contacted the A/STAs’ department at the various healthcare facilities to remind them to complete the questionnaire. One week later, an email reminder via Google forms was sent to the A/STAs’ departments, with a total of three reminders being sent to encourage them to complete the questionnaire in an effort to improve the response rate. The participants were given approximately 3 weeks to complete the questionnaire.

Prior to analysis of the data using the Statistical Package for Social Sciences (SPSS Version 24), the researchers coded the results onto a Microsoft Excel spreadsheet for easy analysis. Once all the data were collected, the results were transferred from Excel to SPSS for analysis. Descriptive statistics and inferential statistics (Pearson’s chi-square test) were used. Information obtained from the open-ended questions was used to report on the participants’ subjective data specifically regarding the implementation of IPC practices within public healthcare facilities in KZN.

To ensure reliability and test the internal consistency of the study, the Cronbach’s alpha was utilised, and revealed a score of 0.821, indicating good internal consistency of the tool (Sirakaya-Turk, Uysal, Hammitt, & Vaske, 2017). The data collection tool was developed based on the findings of previous studies, and an extensive review of the literature was conducted to ensure its reliability and validity (Amlani, 1999; Burco, 2007; Ehlert & Naude, 2014). The content validity of the questionnaire was addressed through the pilot study with six qualified A/STAs. They completed the forms online to test the process. In addition, they had to complete a pilot feedback form indicating the ease of administration, whether the questions were ambiguous, or whether the questions needed to be changed in any way to avoid misunderstanding that could lead participants in a particular direction, thereby creating bias. The changes suggested were implemented in the questionnaire prior to the main study.

### Ethical considerations

Ethical approval was obtained from the Humanities and Social Sciences Research Ethics Committee at UKZN (Ethical clearance number: HSS/0377/017U). Once approval had been obtained from the KZN Provincial Department of Health and Health District Managers, the hospital and medical managers were contacted with a request for permission to conduct the study at the healthcare facilities. Anonymity and confidentiality were maintained, as no names were recorded and the data collected were only available to the researchers, supervisor and statistician. Participation in the study was voluntary and the participants were informed that they could withdraw from the study at any time.

## Results

### Demographic details

Participant demographics are summarised in Table 1, with most being between the ages of 21–30 years, female, having a bachelor degree, with < 6 years’ experience.

**TABLE 1:** Demographic information (*n* = 49).

Category	Sub-category	*n*	%
Age (years)	21–30	38	78
	31–40	9	18
	41–50	2	4
Gender	Male	7	14
	Female	42	86
Years of experience	< 1 year	17	35
	1–5 years	20	41
	6–10 years	8	16
	11–15 years	2	4
	16–20 years	1	2
	> 20 years	1	2
Level of education	Community service	15	31
	Bachelor’s degree	31	63
	Master’s degree	3	6
Practice setting	Rural	21	43
	Semi-rural	9	18
	Urban	17	35
	Semi-urban	2	4
Level of healthcare facility	District hospital	25	51
	Regional hospital	12	25
	Provincial hospital	7	14
	Central hospital	1	2
	Specialised hospital	4	8

The results are presented according to the key objectives of the study as follows:

#### To describe the infection prevention and control policies utilised by audiologists and speech therapists, and audiologists

Sixty-three per cent (*n* = 31) of participants had an audiology-specific IPC policy in place and the majority 82% (*n* = 40) stated that there was a Department of Health (DoH) policy available. Only 25 participants stipulated the type of policy with most 68% (*n* = 17), utilising the National DoH’s IPC policy. Table 2 provides information regarding the participants’ IPC policies used.

**TABLE 2:** Summary of the infection prevention and control policies used by the participants.

Section	Yes		No	
	*n*	%	*n*	%
**Availability of policies (*n* = 49)**				
Audiology infection control policy available	31	63	18	37
Followed a generic (DoH) infection policy	40	82	9	18
**Type of DOH policy followed (*n* = 25)**				
National DOH’s infection control policy	17	68	-	-
Provincial KZN DOH’s infection control policy	2	8	-	-
Hospital infection control policy	6	24	-	-

DOH, Department of Health; KZN, KwaZulu-Natal.

#### To describe the opinions of audiologists and speech therapists, and audiologists regarding the training received and training needed

Only 41% (*n* = 20) of participants received audiology-specific IPC training, with most rating their training as good or satisfactory. In an open-ended response, participants indicated that they would like more information regarding audiology-specific infection control, sterilisation and disinfection of instruments to be included in infection control training. Participants also mentioned that they would like annual education and training sessions (such as workshops, short courses and literature) regarding audiology-specific infection control and that public healthcare facilities should have improved resources for proper implementation of IPC measures. Table 3 provides information regarding training A/STAs received and additional training needs.

**TABLE 3:** Summary of the training the audiologists and speech therapists, and audiologists had received and additional training needs.

Section	Yes		No	
	*n*	%	*n*	%
**Necessity for training and training received (*n* = 49)**				
IPC training was necessary	45	92	4	8
Received audiology-specific training	20	41	29	59
**Participants’ subjective rating of training received (*n* = 20)**				
**Rating**				
Excellent	1	5	-	-
Good	7	35	-	-
Satisfactory	8	40	-	-
Adequate	4	20	-	-
**Additional training needs (number of responses = 85)**				
Audiology-specific IPC measures	22	26	-	-
Sterilisation and disinfection of instruments	21	25	-	-
Personal protective measures	19	22	-	-
Handwashing	12	14	-	-
Waste disposal	6	7	-	-
Advice on ventilation	5	6	-	-

IPC, infection prevention and control.

#### To describe the handwashing practices of audiologists and speech therapists, and audiologists

All of the participants reported that adequate hand hygiene is an essential part of infection control. The results indicated that 98% (*n* = 48) had access to alcohol-based hand rub, and 71% (*n* = 35) had a sink with running water, with the sink being < 50 m from their consultation area. Participants washed their hands with water and soap (33%), hibitane solution (33%) or liquid hand wash (30%). Table 4 illustrates the handwashing practices followed by participants within their working environment. Interestingly, only 76% (*n* = 37) washed their hands prior to managing patients, while the majority 96% (*n* = 47) washed their hands after each patient. Only 67% (*n* = 33) washed their hands after the removal of gloves.

**TABLE 4:** Participants’ handwashing practices.

Handwashing practices	Yes		No	
	*n*	%	*n*	%
Before each patient	37	76	12	24
After each patient	47	96	2	4
After cerumen management	43	88	6	12
After earmould impression taking	47	96	2	4
After the use of the toilet	47	96	2	4
After contact with bodily fluids	46	94	3	6
After removal of gloves	33	67	16	33

*Source:* Authors’ own data compilation with some of the handwashing practices adapted from Amlani (1999), Burco (2007) and Ehlert and Naude (2014)

#### To determine the personal protective measures available and used by the audiologists and speech therapists, and audiologists for infection prevention and control

Eighty-six per cent (*n* = 42) believed that their workplace had a high exposure to communicable diseases, while only 22% (*n* = 11) believed that their workplace had adequate ventilation for individuals with communicable diseases such as TB. Seventy-one per cent (*n* = 35) indicated that masks should always be worn with patients with communicable diseases, while 29% (*n* = 14) felt that this was only necessary sometimes. More than half (57%, *n* = 28) reported wearing gloves sometimes with every patient, while 43% (*n* = 21) always wore them with every patient. An association between the level of the healthcare facility and the wearing of gloves was found to be statistically significant (*p* = 0.025) with more participants at regional and tertiary levels being of the opinion that gloves should be worn during most procedures versus those at district levels of care. Table 5 indicates how often participants wore gloves during various audiological procedures. Participants responded to a three-point Likert scale (with options always, sometimes and never).

**TABLE 5:** Use of gloves during audiological procedures.

Procedures	Always		Sometimes		Never	
	*n*	%	*n*	%	*n*	%
Otoscopy	38	78	11	22	-	-
Immittance	38	78	10	20	1	2
Cerumen management	44	90	4	8	1	2
Evoked potential testing	23	47	23	47	3	6
Vestibular and balance testing	23	47	23	47	3	6
Earmould impression taking	18	36	19	39	12	25
Hearing aid fittings	20	41	20	41	9	18
Hearing aid modifications	20	41	23	47	6	1

*Source*: Authors’ own data compilation with some of the procedures and rating scale adapted from Amlani (1999), Burco (2007) and Ehlert and Naude (2014)

#### To determine the opinions of audiologists and speech therapists, and audiologists regarding equipment management and waste disposal in public healthcare facilities in KwaZulu-Natal, South Africa

**FIGURE 1:**
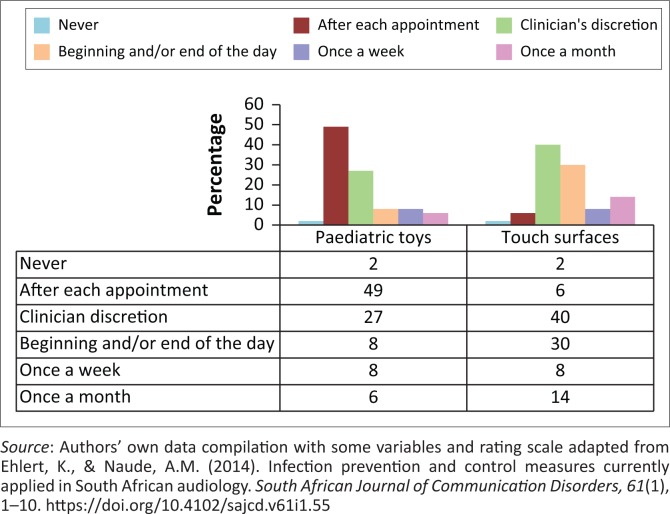
Frequency of disinfecting paediatric toys and touch surfaces (*n* = 49).

All the participants (*n* = 49) contended that the otoscope specula should be disinfected and/or sterilised on a daily basis, while 94% (*n* = 46) and 98% (*n* = 48) indicated that immittance probe tips and headphones should be disinfected and/or sterilised on a daily basis. A few (14%, *n* = 7) believed that earmoulds used during hearing aid fittings should not be disinfected and/or sterilised. The majority of participants contended that otoscope specula, immittance probes, otoacoustic emission (OAE) probes, headphones and insert earphones could expose them to infectious diseases, while 32% (*n* = 16) felt that this was not possible through distraction toys. Only 6% (*n* = 3) believed that touch surfaces, such as countertops, armchair rests or counselling table surfaces, should be disinfected after each appointment. Only 40% (*n* = 19) and 27% (*n* = 13) believed that touch surfaces and paediatric toys, respectively, should be disinfected as needed, based on the discretion of the clinician. Figure 1 illustrates the frequency of participants’ responses in relation to the disinfection of paediatric toys and touch surfaces.

**FIGURE 2:**
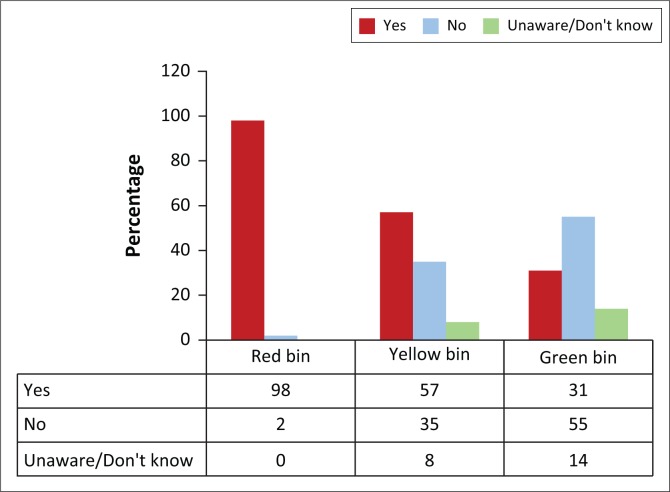
Medical waste disposal bins available at participants’ workplace (*n* = 49).

All the participants believed that medical waste should not be put in the same bin as general waste, and 80% (*n* = 39) indicated that hearing aid batteries should be disposed of separately from other waste. The majority (98%, *n* = 48) indicated that they had red medical waste disposal bins available (for infectious substances such as gloves and alcohol swabs) and 57% (*n* = 28) had the yellow medical waste disposal bins available (for sharps such as needles and syringes), while only 31% (*n* = 15) had the green medical waste disposal bins (for pharmaceuticals, i.e. expired medication) at their workplace (see Figure 2).

## Discussion

Implementing effective infection control measures is essential for any HCP, with adherence to IPC guidelines being stipulated by employing bodies, regulatory bodies (HPCSA, 2002) and professional associations, such as SASLHA (SASLHA, 2011). It was encouraging to note that 67% of the participants had a discipline-specific infection control policy in their workplace, which correlated with results obtained from the study by Burco (2007), who indicated that 61% of respondents had audiology-specific infection control plans in place. Research findings from Burco’s (2007) study indicated that nearly 60% of respondents received audiology-specific infection control training prior to employment, while results from the current research study indicated a lower percentage. It was of concern that some participants were unaware of discipline-specific policies in their workplace. Results from the current study indicated that only 49% believed that paediatric toys should be disinfected after each appointment, while 27% indicated that they would disinfect ‘at the clinician’s discretion’. This correlated with results from Ehlert and Naude’s (2014) study, which indicated that less than half of the respondents disinfected touch surfaces and toys, and would only do so if there was visible contamination. Therefore, participants may have been unwittingly transmitting disease, causing microbes from patient to patient by not adhering to proper infection control guidelines. Education and training for HCPs is essential on a regular basis to increase awareness about infection control policies and guidelines, and it results in improved adherence to these practices and measures, changes negative attitudes and promotes a safer environment (Ojulong, Mitonga, & Iipinge, 2013; Schellack, Ismail, & Babarinde, 2016).

Addis Ababa and India are similar to other developing countries, such as South Africa, where there are minimal efforts to prevent and control HAIs (Sahiledengle et al., 2018). Hand hygiene is the most effective, least costly and simplest method of decreasing the incidence of HAIs (Mathur, 2011) but it is not recognised as such or appreciated by healthcare workers (Ojulong et al., 2013). In healthcare, hands are another extremely mobile surface that are frequently contaminated and seldom disinfected (Peters et al., 2018). All of the participants reported that adequate hand hygiene was an essential part of infection control. The most common handwashing method was washing hands with soap and water and using hibitane, with 33% of participants choosing either method. According to Ehlert and Naude (2014), hand sanitisers are an approved and effective method for hand hygiene, reported to be more effective than washing hands with soap and water. However, according to the WHO’s guidelines on hand hygiene in healthcare (WHO, 2006), based on evidence from several studies, antiseptic alcohol-based hand rubs demonstrated maximum efficacy in removing pathogens from the hands of healthcare workers. These may not be available in all contexts, given costs and cultural beliefs (for example, the Islamic religious beliefs are against the use of alcohol for any purpose). It is thus recommended that currently utilised, commercially available hand rub and liquid soaps meet acceptable standards, as outlined in the guideline for IPC (WHO, 2006).

Almost a quarter of the participants indicated that they did not wash their hands before each patient, which was also a concern raised by Ehlert and Naude (2014); perhaps they believed that they cannot transmit infection to their patients. Approximately one-third of the participants reported not washing hands after removing gloves. Although only 67% of participants believed that it was necessary to wash hands after cerumen management, 88% of participants reported that they washed their hands after cerumen management. However, this is still concerning, as cerumen can be considered an infectious substance and may lead to opportunistic infections, such as methicillin-resistant *Staphylococcus aureus* (MRSA) (Kemp & Bankaitis, 2000; Patel, Engelbrecht, McDonald, Morris, & Smythe, 2016).

Universal precautions are the most effective and simple way of preventing infection in healthcare settings (WHO, 2001). These guidelines include using appropriate PPE or prophylactic measures, such as gloves, N-95 masks (respiratory hygiene), cough etiquette, hand hygiene, environmental cleanliness and waste management (Fayaz et al., 2014; WHO, 2001). It was concerning that more than half of the participants only sometimes wore gloves with every patient, as a 2012 South African survey estimated 12.2% of the population to be living with HIV and AIDS (Shisana et al., 2014). Encouragingly, the majority (78%) of participants were of the opinion that gloves should be worn during routine procedures, such as otoscopy and immittance. One-third felt that gloves should be worn during earmould impression taking and hearing aid fitting, which correlated with results from the study by Ehlert and Naude (2014). These results are concerning as research by Ahmad et al. (2007) indicated that earmoulds may harbour pathogenic microorganisms that can result in the development of chronic otitis externa such as staphylococci, bacteria and fungi (Ahmad, Etheridge, Farrington, & Baguley, 2007; Bankaitis, 2005b).

Half the participants contended that hearing aids could not expose them to infectious diseases, these concerning findings being similar to Bankaitis’s (2002) study, which reported *Staphylococcus* bacterium on the surface of hearing aids, this being implicated in HAIs (Bankaitis, 2002). This was supported in a study by Sturgulewski, Bankaitis, Klodd and Haberkamp (2006), where fungi, bacteria and even faecal matter were found on the surface of hearing aids. Thus, hearing aids have been implicated as a source of microbial transmission and should be treated as a vector (Sturgulewski et al., 2006). Results from Bankaitis (2005b) indicate that as alcohol does not kill bacteria or fungi, given the extent of reported microbial growth on hearing aid and earmould surfaces. Audiologists and speech therapists, and audiologists should be made aware that the use of PPEs is essential while handling hearing aids and all related accessories, as disinfection may be insufficient (Bankaitis, 2005b). Bankaitis and Kemp (2010) suggest that IPC is not an arbitrary process and should be based on established guidelines and compliance thereof.

It was concerning that almost half the participants (47%) indicated that they would only *sometimes* wear gloves during evoked potentials and vestibular testing; however, it was encouraging that an equal number indicated that they would always wear gloves. It was important to note that about a quarter of the participants from Khoza-Shangase and Van Rie’s study (2017) reported that they had diminished hearing sensitivity since being diagnosed with HIV and AIDS. This is further supported by the literature from Heinze, Swanepoel and Hofmeyer (2011), in which reports of auditory and vestibular manifestations were identified in patients with HIV and AIDS (Heinze et al., 2011), necessitating audiological services. Results from the Khoza-Shangase and Van Rie study (2017) also found that patients with HIV and AIDS are prone to middle ear infections and otorrhoea, which are symptoms indicative of middle ear pathology, also present with co-occurring vestibular and audiologic symptoms. As the A/STAs’ scope of practice has increased to include vestibular testing, A/STAs need to be cognisant that some patients who have possible ear infections are likely to be immunocompromised, and that proper use of gloves and other PPEs need to be used during all procedures to prevent the spread of opportunistic infections (Khoza-Shangase & Van Rie, 2017). Many opportunistic infections can be prevented through correct implementation and compliance to evidence-based IPC measures. Although researchers have highlighted the importance of universal precautions, few studies have examined audiologists’ infection control practices, despite the many challenges these HCPs face in resource-limited settings (Russell et al., 2018).

The results from the current study indicated that 25% of the participants believed that masks should only be worn when they are in contact with an individual with a communicable disease. Sahiledengle et al.’s (2018) study revealed that more than half (57.9%) of the HCPs wore masks when in contact with a patient suspected of, or confirmed with having TB, with the results being slightly higher than a study previously conducted in the same city, which revealed that only 50.2% wore masks. However, microorganisms that cause communicable diseases cannot be seen with the naked eye and HCPs would be unaware of the patients’ medical diagnosis (Claassens et al., 2013), leaving them at risk for acquiring a communicable disease. The majority of the participants (86%) reported that their workplace had a high exposure to communicable diseases, which has nearly doubled from Burco’s (2007) and Amlani’s (1999) studies being 48% and 20%, respectively. Seventy-eight per cent of the participants reported that their workplace did not have adequate ventilation, this being important for audiologists whose patients present with TB for ototoxic monitoring (Claassens et al., 2013).

Encouragingly, results from the current research study indicated that all participants believed that otoscope speculae should be sterilised on a daily basis, which is an improvement from Burco’s (2007) study, where 42% of respondents indicated that they sterilised and/or disinfected otoscope. Research indicates that the practices of sterilisation and disinfection of audiological equipment have steadily improved over the years. In the current study, the participants did not believe that hearing aids or acrylic moulds could expose them or their patients to infectious disease, warranting further education and training (SASLHA, 2011). According to Bankaitis and Kemp (2002), whether attempting to obtain an earmould impression, using the earmould when doing a hearing aid fitting or removing the earmoulds from the patients ear (e.g. making adjustments to the hearing aid), it must be cleaned and handled with gloves. The earmould makes contact with the patient’s ear canal and can be contaminated with substances lining the ear canal with blood, cerumen or ear infection. It is therefore important to use a disinfectant spray on the hearing aid and earmould to minimise the risk of cross-infection. Implementing effective IPC practices in the audiological environment also provides an opportunity for A/STAs to educate their patients about proper hearing instrument hygiene, care and maintenance (Bankaitis, 2002).

Equipment and surfaces that may be frequently touched are thought to provide the greatest risk for patients (Dancer, 2009). In Burco’s study, one-third of the participants did not believe that distraction toys could expose them to infectious disease (Burco, 2007). The results regarding disinfecting at the clinician’s discretion correlated with Burco’s (2007) findings, which indicated that 40% would only disinfect when there was visible contamination. Paediatric toys and touch surfaces should be sterilised according to professional infection control guidelines and after each patient appointment to prevent cross-contamination (Dancer, 2009).

Research regarding audiology-specific waste disposal methods is minimal, and according to universal precautions and infection control guidelines, infectious waste should be disposed of appropriately (SASLHA, 2011). It was encouraging to note that all the participants believed that medical and other waste should be disposed of separately, as medical waste can cause the possible spread of disease if it is not disposed of properly. Fourteen per cent of the participants believed they should only ‘sometimes’ follow the correct medical waste disposal methods, which may have been because of their not having the correct medical waste disposal bins available. This correlated with results from the current study, which indicated that 35% of the participants did not have the yellow and 55% did not have the green medical waste disposal bins in their workplace.

## Conclusion

The study aimed to determine the IPC measures and opinions of A/STAs in public healthcare facilities in the KZN province, South Africa. The self-reported findings suggest variable practices and mixed opinions about IPC practices. Despite training and the availability of policies, some participants displayed poor adherence to standard IPC practices, while others indicated the non-availability of policies and/or discipline-specific training. This suggests that audiology clinics need to be equipped with adequate discipline-specific policies, and that A/STAs be trained on IPC issues relevant to their practice. Hand hygiene is a cost-effective intervention that should be practiced before and after patients, even if gloves were worn. Poor compliance to IPC measures may stem from a deficit in the knowledge and/or attitudes, including perceived barriers such as lack of time, patient and HCP discomfort, lack of resources (PPE) and the sense that protective equipment interferes with work performance. An effective infection control programme depends largely on the personnel responsible for its implementation, there has to be a mechanism in place for monitoring. Information, education, training, guidelines and effective practices are essential to break the cycle of inadequate infection control. Ultimately, it is the audiologist’s responsibility to ensure that effective IPC measures are routinely adhered to in clinical practice.

## Limitations and recommendations

A small sample size limited the generalisability of the study to HCPs, as it was only conducted in public healthcare facilities in KZN. Information bias may have occurred as participants may not have taken time to consider each question, but may rather have completed the questionnaires quickly. The compliance with IPC was based on reported practices of audiologists and not those directly observed by the researchers, which could have resulted in them providing answers that they thought more appropriate, thus introducing social desirability bias. The researchers could have probed more about the barriers to IPC practices that would have been useful when suggesting further clinical and research implications. Increasing the awareness of the importance of IPC within healthcare facilities will help motivate HCPs to implement adequate measures throughout their clinical practice and ensure compliance. Healthcare practitioners could also counsel their patients about the proper cleaning and disinfecting of their hearing instruments. Future research could venture into a more in-depth investigation and consideration of the real environment of audiological practice in public hospitals for more meaningful recommendations to be made. The effectiveness of IPC measures in audiology needs further investigation. The profession needs to advocate for IPC resources to decrease the risk of infections and cross-contamination in healthcare facilities.

## Appendix 1

### Questionnaire

#### Section A: Demographical information

**(Please circle or tick the option(s) that fit best)**

**Table:**

Location: KwaZulu-Natal						
1. Age (years)	21–30	31–40	41–50		51–60	60+
2. Gender	Male			Female		
3. Years in practice	<1 year	1–5 years	6–10 years	11–15 years	16–20 years	>20 years
4. Level of education	Community service	Bachelor’s degree		Master’s degree	Doctoral	

**Table:**

5. In what setting do you practice?	
Rural	
Semi-rural	
Urban	
Semi-urban	

**Table:**

6. What is the level of the healthcare facility that you currently work at?	
District hospital (level 1)	
Regional hospital (level 2)	
Provincial tertiary hospital (level 3)	
Central hospital (level 4)	
Specialised hospital	

**Table:**

7. Which population do you provide services to at your current workplace?	
Only adolescents or adults	
Majority adolescents or adults	
Fairly balanced number of adolescents or adults (50%) and paediatrics (50%)	
Majority paediatrics	
Only paediatrics	

**Table:**

8. Please list the top five populations which you provide services to in your current workplace (e.g. TB adults, CP children).			
Q8 a)	Children:	Q8 b)	Adolescents or adults:
			
			
			
			
			

**Table:**

9. In general, which services do you provide to patients? (please tick the option[s] that apply)			
Q9 a)	Otoscopy		
Q9 b)	Immittance audiometry		
Q9 c)	Pure tone audiometry		
Q9 d)	Otoacoustic emissions		
Q9 e)	Auditory brainstem response		
Q9 f)	Auditory steady-state response		
Q9 g)	Electronystagmography (ENG) or videonystagmography (VNG) (vestibular and balance testing)		
Q9 h)	Hearing aid fitting and orientation		
Q9 i)	Central auditory processing		
Q9 j)	Cerumen management		
Q9 k)	Aural rehabilitation		
Q9 l)	Other (please specify)		

#### Section B: Infection prevention and control policies and training

**Table:**

10. Do you follow a specific infection control policy (i.e. National Infection Control Policy) at your workplace?	
Yes	
No	
Unaware or don’t know	

**Table:**

11. Do you have a discipline-specific infection control policy or guideline in your professional setting?	
Yes	
No	
Unaware or don’t know	

**Table:**

12. In your opinion, should you receive undergraduate training in infection control?	
Yes	
No	
Unaware or don’t know	

**Table:**

13. Have you received any training on audiology-specific infection control in your workplace?	
Yes	
No	
Unaware or don’t know	

If you answered yes to the above question, please answer the question below.

**Table:**

14. How would you rate the audiology-specific infection control training you received?	
Excellent	
Good	
Satisfactory	
Adequate	
Poor	

**Table:**

15. In your opinion, do audiologists need to attend any educational workshops, short courses and so on, regarding infection control practices?	
Yes	
No	
Don’t know	

**Table:**

16. In your opinion, would attendance to infection control workshop(s) influence your infection control measures at your workplace?	
Yes	
No	
Don’t know	

**Table:**

17. In your opinion, list content areas you feel should be included in infection prevention and control training for audiologists.

#### Section C: Handwashing

**Table:**

18. Do you follow the following procedures? (Please tick the option[s] that fit best)				
		Always	Sometimes	Never
Q18 a)	Washing your hands			
Q18 b)	Wearing gloves with every patient			
Q18 c)	Wearing masks with patients with a communicable disease			
Q18 d)	Correct medical waste disposal			
Q18 e)	Sterilising equipment			
Q18 f)	Disinfecting equipment			

**Table:**

19. Do you have access to alcohol-based hand rub at your workplace?	
Yes	
No	
Unaware or don’t know	

**Table:**

20. Do you have a sink with running water in your consultation area?	
Yes	
No	

**(If you answered yes to the above question, please answer the question below)**

**Table:**

21. What is the distance of the sink in relation to your consultation area?	
<50 m	
50–100 m	
>100 m	
	

**Table:**

22. In your opinion, should hands be washed … ? (Please select those which apply to you)		
Q19 a)	Before each patient	
Q19 b)	After each patient	
Q19 c)	After cerumen management	
Q19 d)	After earmould impression taking	
Q19 e)	After use of the toilet	
Q19 f)	After contact with bodily fluids	

**Table:**

23. Do you think adequate hand hygiene is an essential part of infection control?		
Always		
Sometimes		
Never		
Not applicable		
If you selected ‘sometimes’ please explain when …		

**Table:**

24. Which of the following do you consider as adequate hand hygiene? (Please tick the option/s that apply)		
Q21 a)	Washing your hands after removal of gloves?	
Q21 b)	Washing your hands after cerumen management?	
Q21 c)	Washing your hands before contact with every individual patient?	
Q21 d)	Washing your hands after contact with each individual patient?	

**Table:**

25. Do you wash your hands with … ?	
Water	
Water and soap	
Hibitine solution	
Liquid hand wash	
Other:	

#### Section D: Personal protective measures

(Please tick the option[s] that fit best)

**Table:**

26. Should audiologists receive vaccinations for hepatitis B?	
Yes	
No	
Unaware or don’t know	

**Table:**

27. Do you think your workplace has a high exposure to communicable disease(s)?	
Yes	
No	
Unaware or don’t know	

**Table:**

28. Do you think your workplace has adequate ventilation for those individuals who may have communicable diseases (e.g. TB)?	
Yes	
No	
Unaware or don’t know	

**Table:**

29. In your opinion, should masks be available at your workplace?	
Yes (always available)	
Sometimes	
No (never)	

**(If you answered ‘yes’ to the above question, please answer the question below)**

**Table:**

30. In your opinion, when should masks be worn?	
With every patient	
Sometimes	
Only when in contact with a person with a communicable disease (e.g. TB)	

**Table:**

31. In your opinion, when should gloves be worn, during … ? (Please tick the option that applies in each case)				
		Always	Sometimes	Never
Q31 a)	Otoscopy			
Q31 b)	Immittance			
Q31 c)	Cerumen management			
Q31 d)	Evoked potentials			
Q31 e)	Vestibular and balance testing			
Q31 f)	Earmould impression taking			
Q31 g)	Hearing aid fittings			
Q31 h)	Hearing aid modifications			

**Table:**

32. Have you sustained any injuries or infections at your workplace related to poor infection prevention and control measures? If so please explain …

#### Section E: Management of equipment and waste disposal

**(Please tick the option[s] that fit best)**

**Table:**

33. In your opinion, on a daily basis, should the following instruments be disinfected and/or sterilised after use? (Please tick all the option/s that apply)		
Q33 a)	Otoscope specula	
Q33 b)	Reusable auditory brainstem response (ABR) electrodes	
Q33 c)	Reusable electronystagmography (ENG) electrodes	
Q33 d)	Otolight	
Q33 e)	ENG irrigator tip	
Q33 f)	ENG specula	
Q33 g)	Real-ear probe tips	
Q33 h)	Headphones or insert earphones	
Q33 i)	Immittance probe tip	
Q33 j)	Acrylic earmoulds used during hearing aid fitting	

**Table:**

34. Which items do you think could most likely expose one to infectious diseases?		
Q34 a)	Otoscope specula	
Q34 b)	Immittance probes	
Q34 c)	Hearing aids	
Q34 d)	Otoacoustic emission (OAE) probes	
Q34 e)	Headphones and insert earphones	
Q34 f)	Distraction toys	

**Table:**

35. In your opinion, how often should the following activities be conducted at your current workplace?					
		Always	Sometimes	Never	N/A
Q35 a)	Sterilise equipment				
Q35 b)	Clean the audiometer				
Q35 c)	Disinfect headphones used during Pure Tone Audiometry (PTA)				
Q35 d)	Sterilise otoscope specula				
Q35 e)	Sterilise probe tips used				

**Table:**

36. In your opinion, how often are touch surfaces (i.e. countertops, counselling table or armchair rests) disinfected at your place of work?	
Never	
After each patient appointment	
As needed based on the discretion of the clinician	
At the beginning and/or end of the day	
Once a week	
Once a month	
Unaware or don’t know	
Other (e.g. responsibility of the cleaning staff)	

**Table:**

37. In your opinion, how often should toys used during paediatric hearing assessments be disinfected?	
Never	
After each patient appointment	
As needed based on the discretion of the clinician	
At the beginning and/or end of the day	
Once a week	
Once a month	
Not applicable	
Don’t see paediatric patients	

**Table:**

38. Are there medical waste disposal bins available in your current work setting?				
		Yes	No	Unaware or don’t know
Q38 a)	Red (infectious substances, i.e. gloves, swabs)			
Q38 b)	Yellow (‘sharps’, i.e. needles, syringes)			
Q38 c)	Green (pharmaceuticals, i.e. expired medication)			

**Table:**

39. In your opinion, should medical waste be put in the same dustbin as general wastes?	
Yes	
No	
Unaware or don’t know	

**Table:**

40. In your opinion, should hearing aid batteries be disposed of separate from other wastes?	
Yes	
No	
Unaware or don’t know	

**Thank you for your time and participation.**
